# A direct comparison of sound and vibration as sources of stimulation for a sensory substitution glove

**DOI:** 10.1186/s41235-023-00495-w

**Published:** 2023-07-04

**Authors:** Carlos de Paz, David Travieso

**Affiliations:** Facultad de Psicología, Universidad Autónoma de Madrid, 28049 Madrid, Spain

**Keywords:** Sensory substitution devices, Multimodal, Grasping, Visual impairments

## Abstract

Sensory substitution devices (SSDs) facilitate the detection of environmental information through enhancement of touch and/or hearing capabilities. Research has demonstrated that several tasks can be successfully completed using acoustic, vibrotactile, and multimodal devices. The suitability of a substituting modality is also mediated by the type of information required to perform the specific task. The present study tested the adequacy of touch and hearing in a grasping task by utilizing a sensory substitution glove. The substituting modalities inform, through increases in stimulation intensity, about the distance between the fingers and the objects. A psychophysical experiment of magnitude estimation was conducted. Forty blindfolded sighted participants discriminated equivalently the intensity of both vibrotactile and acoustic stimulation, although they experienced some difficulty with the more intense stimuli. Additionally, a grasping task involving cylindrical objects of varying diameters, distances and orientations was performed. Thirty blindfolded sighted participants were divided into vibration, sound, or multimodal groups. High performance was achieved (84% correct grasps) with equivalent success rate between groups. Movement variables showed more precision and confidence in the multimodal condition. Through a questionnaire, the multimodal group indicated their preference for using a multimodal SSD in daily life and identified vibration as their primary source of stimulation. These results demonstrate that there is an improvement in performance with specific-purpose SSDs, when the necessary information for a task is identified and coupled with the delivered stimulation. Furthermore, the results suggest that it is possible to achieve functional equivalence between substituting modalities when these previous steps are met.

## Significance

The development of assistive technologies in the form of sensory aids has been a major applied goal since the deployment of the first sensory substitution devices (SSDs) in the 1960s. Sensory substitution devices attempt to substitute vision by delivering sensory information through hearing or touch or by enhancing their capabilities. The fact that SSDs allow for an independent analysis of sensory modalities, perceptual information, and sensorimotor contingencies, gives them a privileged status for the empirical analysis of sensory modalities and multimodal integration.

The question of which substituting modality is more suitable for SSDs has yet to be answered. When comparing sensory modalities, it is important to consider the type of information required to complete the task and whether the substituting modality is suitable for delivering that information. Consequently, this field of research would benefit from a wider corpus of performance comparisons between different sensory modalities with constant elements of the SSDs.

In this study, a fair comparison of hearing and touch as substituting modalities in a sensory substitution glove that enables the grasping function. The substituting modalities inform, through increases in stimulation intensity, about the distance between the fingers and objects. The results revealed high equivalent success rate between sensory modalities, and a multimodal condition including both. Sensory modalities were equipotent when they deliver intensity variations in correspondence with distance information, under similar sensorimotor contingencies. Furthermore, we interpreted multimodal integration to depend as being more on these contingencies than on a basic compatibility between sensory modalities.

## Introduction

Sensory substitution devices (SSDs) are electronic devices that facilitate the detection of environmental information through enhancement of touch and/or hearing capabilities. These devices have been shown to be effective in tasks such as object and pattern recognition (Auvray et al., 2007; Bach-y-Rita et al., 1969; Bermejo et al., 2015; Kaczmarek & Haase, 2003; Rovira et al., 2010; Sampaio et al., 2001), obstacle avoidance during navigation (Chebat et al., 2011, 2015; Froese et al., 2011; Kilian et al., 2022; Kolarik et al., 2017; Lobo et al., 2018, 2019; Maidenbaum et al., 2014b; Starkiewicz & Kuliszewski, 1963), and some forms of affordance-based perception (De Paz et al., 2019; Favela et al., 2018; Kolarik et al., 2014; Travieso et al., 2015).

The primary applied goal of SSDs is the rehabilitation of individuals with visual impairments. However, despite their potential, these devices have not yet achieved widespread use among this population (Elli et al., 2014; Maidenbaum et al., 2014a; Spence, 2014). Autonomous mobility is the most reported problem among people with visual impairments, and most SSDs are designed to improve this ability, including wayfinding (orientation) and navigation (obstacle avoidance). Nevertheless, it is important to note that another specific impairment resulting from the absence of sight is the difficulty in performing daily activities, particularly the localization of objects in peripersonal space that are not in direct contact with the individual (Castiello et al., 1993). Thus, allowing for the localization, reaching, and grasping of objects in peripersonal space is also an important applied goal for SSDs.

The selection of a perceptual substitution system is not a simple decision in sensory substitution. According to Auvray et al. (2007), there are more haptic devices available than acoustic ones. However, when choosing a substitution system, authors tend to use practical rather than theoretical or efficiency and effectiveness considerations. Those who advocate for the use of haptics argue that: (1) touch is an underutilized sense, whereas hearing is essential in many daily tasks and its usability can be reduced or blocked by the SSD; (2) different types of stimulation (e.g. vibration, electricity) can be used (Kaczmarek et al., 1991); (3) actuators can be placed on various parts of the body, including non-visible locations such as the abdomen, allowing the device to be hidden (Barontini et al., 2020; Spence, 2014; Visell, 2009). Conversely, those who favor the use of hearing argue that: (1) the use of the skin has drawbacks such as irritation, pain, and poor discrimination; (2) acoustic devices have a simpler interface (e.g. headphones); (3) they require less energy; (4) digital sound processing is a widely available technology (Auvray & Myin, 2009; Auvray et al., 2007; Capelle et al., 1998).

A theoretical approach to select the substituting sensory modality and specific stimulation is proposed by Loomis et al. (2012). They emphasized how general-purpose SSDs have failed to fully substitute vision. On the other hand, more promising results have been achieved with special-purpose devices designed to fulfill specific sensorimotor functions. Therefore, they recommended a two-step process when designing an SSD. The first step is to identify the necessary environmental information to solve the task, which they call the informational requirements. The second step is to deliver that information through stimulation via the substituting modality, which they call coupling task information with the substituting modality. Thus, the selection of the substituting modality should be based on the feasibility of its sensory bandwidth, the need for higher-level processing, and the need for spatial isomorphism with the environmental information, if required.

According to Lenay et al. (2003), there is no a priori reason to expect any dominance in performance between different substituting modalities, as the brain's plasticity is believed to be equipotential for various modalities. Moreover, Lenay et al. highlighted that certain features can be perceived through sensorimotor contingencies, that is, through sensory information coupled to the movements of the perceiver. For these features, one would expect functional equivalence between sensory modalities when sensorimotor contingencies yield equivalent information. Furthermore, Lloyd-Esenkaya et al. (2020) suggested that touch and hearing can be interchangeably used to provide similar types of information, as demonstrated by several multimodal studies involving both haptic and acoustic devices, such as the EyeCane (Amedi et al., 2011; Maidenbaum et al., 2014a) and the Sound of Vision (SoV) (Hoffmann et al., 2018). An important implication of the above theories is that the possibility of multimodal integration would depend on the information provided to different substituting modalities. Loomis et al. (2012) argued that the multimodal integration would depend on the construction of amodal representations of the task, which is in line with redundancy theories (Mayer & Johnson, 2008) that predict better performance when similar information is obtained at the same time during the task. However, certain authors argue for the use of one specific modality over another. Spence (2014) highlighted that touch is spatiotopic, similar to vision, and thus more suitable for conveying spatial information than hearing, which is tonotopic. However, it is important to highlight that hearing has also a spatial pathway through the superior olivary that integrates interaural differences (Oldfield & Parker, 1984), and allows spatial localization (Jenkins & Masterton, 1982) and the use of spatialized audio. Conversely, Auvray et al. (2007) claimed that hearing has better frequency and intensity resolution, making it more suitable for dealing with rapid changes and noisy environments.

Comparing haptic and acoustic SSDs is challenging due to multiple confounding factors (Richardson et al., 2019). These include differences in the type of information provided by the SSDs, as well as variations in the settings and tasks for which the devices are used, making it difficult to perform systematic reviews or meta-analyses. The exception to this is visual acuity tests (Sampaio et al., 2001), in which standardized ophthalmological tests (i.e., Snellen E optotype) are used to measure the performance of the SSD. However, even in these tests, differences in exploratory patterns are not controlled.

One way to study the differences in substituting modalities is to directly compare the performance achieved using different SSDs on the same task. An example of these comparisons is Kolarik et al. (2017), who compared echolocalization with a tactile SSD in a circumvention task. However, as Richardson et al. (2019) highlighted, it is essential to control for confounding factors across modality conditions to ensure fair comparisons. Recently, Jicol et al. (2020) conducted an experiment that compared the performance of two SSDs, an acoustic one (the vOICe, Meijer, 1992) and an electrotactile one (the BRAINPORT, Bach-y-Rita & Kercel, 2003), in a navigation task. The task involved exploring an aerial map of a 3D setting using the SSDs and then walking to targets in the real setting without the stimulation of the SSDs. Movement data were collected using a motion tracking system, and end positions (constant and variable errors) were recorded. The results indicated that there were no significant differences in performance between the two devices alone, and both showed a similar learning effect (error reduction over trials).

In addition, Jicol et al. (2020) reported that when the acoustic and haptic SSDs were combined, users did not show an improvement in performance. The authors suggested that this could be due to not only the differences in sensory modalities but also to the differences in the information provided. The vOICe converts the image by scanning the environment, so the higher luminance of the pixel the louder the sound. Furthermore, pixels are played sequentially from left to right, and the pitch lowers progressively from top to bottom of the image. On the other hand, the BRAINPORT transforms the image into a matrix of actuators in 2D correspondence, where the brighter areas in the image are represented by pulses of higher voltage levels. Therefore, the authors hypothesized that the lack of multimodal integration may be due to cognitive overload when interpreting both types of information simultaneously. Similarly, Stein and colleagues (Stein, 1998; Stein & Wallace, 1996; Stein et al., 2010) pointed out that the sensory cues of each modality might not have been integrated into a single multisensory stimulation but were detected as two independent unisensory sources of information.

Another study that compared tactile and acoustic SSDs is Richardson et al. (2019). They examined the spatial perception of depth and height using three sensory modalities (i.e., visual, acoustic, haptic) in a two-alternative forced choice task with a staircase procedure. The SSDs had the same distance range (2 m) and the same linear transformation of distance into stimuli intensity (i.e., changes in lightness, loudness, and vibrotactile amplitude, respectively). However, while the spatial stimulation (i.e., the number of points in the stimulation matrix) was the same for the visual and vibrotactile devices, the acoustic SSD delivered a sonification of the depth map. The results showed that there were no significant differences in height discrimination between the acoustic and haptic SSDs, while the acoustic SSD outperformed the tactile SSD in depth perception. However, participants were constrained in their range of movements, and while the devices had the same resolution, they delivered different information concerning depth.

On the other hand, positive cases of using acoustic and tactile SSDs in conjunction have been described in Hoffmann et al. (2018) using the SoV and in Maidenbaum et al. (2014b) using the EyeCane (Amedi et al., 2011). Both studies involved participants performing a navigation task using SSDs that provide acoustic and haptic stimulation on the distance to the first-encountered object. However, neither study compared the different sensory modalities separately, nor did they manipulate the stimulation to control whether participants ignored one source of information.

The primary objective of this study is to compare vibrotactile and acoustic sources of stimulation, in accordance with the design criteria proposed by Loomis et al. (2012). Specifically, we aim to use a SSD that detects the function-relevant information and delivers detectable stimulation lawfully coupled to user exploration. To accomplish this, we selected a grasping task that had previously been studied in De Paz et al. (2023). The initial step was to determine the information required to perform the grasping behavior, which would be delivered via the SSD.

Two models have been proposed to explain how we grasp objects under visual control. The double-pointing model (Smeets & Brenner, 1999; Verheij et al., 2012) posits that grasping consists of directly moving the fingers to the appropriate positions on the surface of the object. The visual information needed for the grasping function is the localization of the target positions on the object. On the other hand, the visuomotor channels model (Jeannerod, 1984, 1999) suggests that prehension is composed of two distinct phases. After detecting the object to be grasped, we first approach it, using a ballistic movement of the hand (the reaching component). This phase is dependent on the visual localization of the object in peripersonal space. Once the object is approached, we begin to open the hand by increasing the distance between the index finger and the thumb until both fingers are positioned on the surface of the object. This second phase is guided by the visual detection of the object's intrinsic properties, such as size and shape. More importantly, despite differences in the motor control models, both approaches agree in that the necessary information to perform the task is the object relative distance and orientation, as well as its size (to control the opening of the hand) and/or the detection of object edges (to place the opposing fingers).

Since SSDs are designed for people with visual impairments, it is important to consider non-visual grasping characteristics. However, there are no alternative models of non-visual grasping in terms of the information needed to perform the task. Castiello et al. (1993) studied a grasping task with blind participants who first explored the object with one hand and, afterward, grasped it with the other hand. The study showed that there were no fundamental differences in grasping behavior between people with visual impairments and sighted blindfolded participants, but longer movement times and larger hand apertures. Similar results were observed when sighted participants were allowed to see the object and then grasped it with their eyes closed (Jakobson & Goodale, 1991; Wing et al., 1986).

A comparison of a grasping task performed through a SSD delivering acoustic (verbal) or vibrotactile information was performed by Mante and Weiland (2018). Their device consists of a camera system mounted on spectacles, capable of locating the position of an object in the visual field. The feedback informs about the direction of the movement that should be made with the head in order to focus the object on the center of the visual field. The feedback is verbal in the acoustic condition (i.e., “move up” or “move down”) and through a 2 × 2 matrix of spatially distributed positions for vibration (i.e., the two left motors vibrate, so move left). No depth information was given. Once the object was centered, users had to guide the hand toward the object by establishing the proprioceptive relation of hand position and head orientation. There were no significant differences in performance between sensory modalities but larger exploration for the vibrotactile condition. Participants needed around 20 s to locate the object at the center of their visual field. In addition, no reports of the amount of correct grasping or number of attempts were reported.

De Paz et al. (2023) demonstrated that by providing vibrotactile distance information to the object in the direction of pointing of the thumb and index finger, it is possible to accurately grasp objects of varying sizes, at different distances, and orientations. In this way, participants could access both the size and the edges of the object, in addition to the vibrotactile distance information, by using proprioceptive information about their finger positions while pointing to the object. With the use of the glove, the grasping behavior exhibited three distinct phases: first, users detect the object's orientation through wrist and arm rotations. Then, users transport the hand toward the object, and finally, they open the hand and grasp the object. Consequently, in addition to the reaching and grasping phases, described under visual control models, an exploration/localization phase also appears.

As already mentioned, this device conveys information about the first object encountered in the pointing direction of the index finger and thumb through changes in stimulation intensity. The selection of intensity as the parameter of interest derives from the need to meet the two-steps criteria of Loomis et al. (2012), that is, to inform about the distance to the object and to do so through changes in magnitude that can be perceived equivalently in the compared sensory modalities. Other types of information and signal parameters could be used, such as beats of increasing frequency or changes in pitch, but thanks to the work of Loomis et al. (2012) we already know that these parameters do not generate equivalence between sensory modalities. Thus, gradients linked to spatial properties would introduce significant differences between modalities (Loomis et al., 2012). The frequency parameter produces significant differences in vibrotactile perception and acoustics, giving raise to the perception of pitch, whose functioning strongly differ from that of vibrotactile frequency.

In the present study, the tactile version of the device utilizes vibration motors on each finger, while the acoustic version employs an acoustic signal to the ipsilateral ear (left-thumb and right-index finger for a right-hand grip). Two experiments were conducted in this study. Experiment 1 utilized a magnitude estimation psychophysical task to evaluate potential differences in sensation levels between the two sensory modalities as a function of equivalent changes in intensity. Experiment 2 involved a real grasping task. The performance of this full sensorimotor task, which is essential for the main purpose of SSD design and testing, and the fact that it has not been systematically studied with acoustic devices, highlights the importance of this research.

## Experiment 1: psychophysical task

In order to make a fair comparison of performance in a full sensorimotor task, it is necessary to first determine the extent to which users perceive intensity increments in touch and hearing equivalently. The most appropriate method for achieving this is to ensure that the intensity range and levels are both detectable and discriminable (García-Valle et al., 2020). By doing so, it is possible to produce a fair comparison in a full sensorimotor task that is controlled by information that is equivalent in perceptual terms.

Changes in intensity in both modalities must be well-detected in order to correctly perform the task. Additionally, multisensory integration (Kayser & Logothetis, 2007; Lloyd-Esenkaya et al., 2020) implies that changes in both sensory modalities should be distinguishable at the same time. If this is the case, we can access the same information through two modalities that do not compete, but rather complement each other. Multisensory integration has not always been achieved with SSDs. While in Maidenbaum et al. (2014a) users did not report feeling overloaded by using vibration and sound simultaneously, it did happen in Jicol et al. (2020). In Jicol et al. (2020) users may have not been able to integrate both sources of information as they may have not been compatible, which would require participants to perform extra mental activities.

In light of these considerations, the aim of this first experiment is to test whether participants attribute similar levels of sensation to two sensory modality stimuli in a psychophysical magnitude estimation task. It is important to highlight that the experiment is not intended to match the subjective intensity of the signals, but to detect the range of increments equivalently in both modalities. Stimuli for the haptic modality were vibrotactile stimulation varying in intensity as a function of the voltage submitted to the motors, whereas it was the amplitude of a pure tone for hearing. We analyze if the selected levels, ranging from mild to moderately high intensities in each continuum, produce similar psychophysical fits for both modalities. We also hypothesize that increasing magnitudes would result in a monotonic trend in the sensation levels for both modalities.

### Method

#### Participants

Forty participants (four males) with an average age of 19.82 years old (SD = 2.54) performed Experiment 1. All participants were right-handed, psychology students and had no prior experience with SSDs. Informed consent was obtained from all participants and the experimental protocol was approved by the local ethics committee. Participants received course credit for their participation.

#### Apparatus

The present study utilized the same sensory substitution glove as previously described by De Paz et al. (2023), consisting of a polyester glove and two vibrotactile coin motors located on the back of the second phalanx of the index finger and thumb. Although the hairy skin of the back of the fingers has a tactile spatial acuity of an order lower than the finger pads (Verrillo, 1966), and there are guidelines for implementing vibrotactile stimulation on the finger pads (Gorlewicz et al., 2020), the grasping function requires the inner part of the hand to be clean for appropriate grasp. Tactile contact of the glabrous skin has been shown to play a critical role in haptic perception (Ellis & Lederman, 1999; Flanagan & Wing, 1997; Lederman & Klatzky, 1987). In addition, the differences in sensitivity of hairy versus glabrous skin for vibrotactile stimuli is not as profound as for spatial acuity. Mahns et al. (2006) showed that, despite different thresholds, frequency discrimination had less differences and was even similar at the fingertips and forearm for frequencies below 50 Hz.

These motors were connected to a computer (PC Intel Core i7, 3.07 GHz) via a printed circuit board (PCB) to display vibrotactile stimulation. Acoustic stimulation was delivered via headphones. The intensity of stimulation was calculated as follows: (1) for vibrotactile stimulation, the range of intensities used in De Paz et al. (2023) from 3.1 to 10 V was employed, as these were the minimum and maximum voltages that our vibrating motors could display; (2) for the acoustic signal, a 200 Hz pure tone was used, with minimum and maximum sound levels set at 45 and 83.88 dB, respectively, as these values correspond to moderate to the maximum dB below a noise dose. The stimulation intensity was controlled using a self-developed routine in MATLAB (The MathWorks, Inc., Natick, 2016).

#### Design

A psychophysical magnitude estimation task was employed in which participants were asked to numerically report the perceived intensity of a predefined set of stimuli by comparing them to a reference magnitude with an arbitrary value of 50. Two independent variables were included as within-subject factors. The first was the type of stimulation (vibration or sound), with the order of sensory modality counterbalanced. The second independent variable was the intensity factor. To select the stimulation levels, the range of stimulation intensity was split into 11 equal steps, as shown in Table 1. The reported perceived intensity for each stimulus was the dependent variable. Each condition was repeated five times in a randomized order, resulting in 120 experimental trials in total (10 intensities × 5 repetitions × 2 modalities + 20 reference trials).

**Table 1:** The intensity of the 11 stimuli

	1	2	3	4	5	6	7	8	9	10	11
Voltage	3.1	3.79	4.48	5.17	5.86	6.55	7.24	7.93	8.62	9.31	10
dB	45	48.89	52.77	56.66	60.55	64.33	68.33	72.22	76.11	79.99	83.88

Intensity of each stimulus. The vibration range went from 3.1 to 10 V (0.69 V increments), while the sound range went from 45 to 83.88 dB (3.89 dB increments). The sixth stimulus was used as reference

#### Procedure

Participants were informed that they would be completing a magnitude estimation task, which involves assigning numerical values to the perceived intensity of a signal based on a reference stimulus. In this study, the sixth step of intensity was used as the reference, with an arbitrary value of 50. The reference stimulus was presented every five trials, and participants were informed that it was a reference trial and had an intensity value of 50. After the presentation of each stimulus, participants were asked to report their perceived level of sensation. Each stimulus was presented for 0.5 s. Prior to the experimental trials, each stimulation level was displayed in a randomized order without feedback. The experiment was conducted in a 30-min session.

#### Statistical analysis

We performed two statistical analyses. The first test was an equivalence Bayesian dependent samples to test whether the perceived intensity with each sensory modality group was not significantly different from the other group. Then, we carried out a repeated-measures analysis of variance (ANOVA) on the level of sensation reported by participants, with the 10 experimental intensities and the two modalities as within-subject factors. When the assumption of sphericity was violated, Greenhouser-Geisser corrections were applied. The level of significance was set at *α* = 0.05. As a post-hoc analysis, the Bonferroni correction test was used. Best fits between the objective and estimated intensities were also obtained.

### Results

**Fig. 1:**
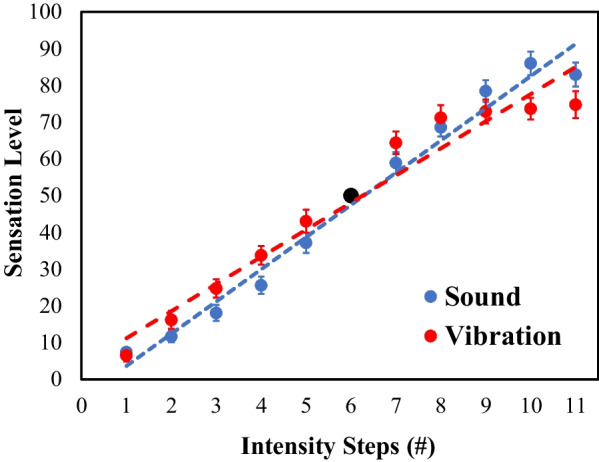
Sensation levels for each sensory modality as a function of the intensity steps. *Note*. Mean numerical estimates of sensation as a function of intensity level for vibration (in red) and sound (in blue) in the magnitude estimation task. The sixth step (black dot) was used as reference intensity and was given a value of 50 (arbitrary scale). The dashed lines represent the best fits to the data

The equivalence test revealed, with moderate evidence (BF_10_ = 5.14), that the two sensory modalities increments received equivalent estimations (Sound: *M* = 47.54, SD = 7.78; Vibration: *M* = 48.01, SD = 9.05). Then, we performed a repeated-measures ANOVA to study the effect of the intensity factor and its interaction with the sensory modality. There was a strong and significant effect of the intensity factor, *F*(3.06, 351) = 956.99, *p* < 0.001, *n*_*p*_^2^ = 0.96, indicating that as the intensity increased, the level of sensation reported by participants also increased. The Bonferroni post-hoc test revealed that each step of intensity was significantly different from the others (*p* < 0.001). Moreover, there was a significant interaction effect, *F*(4.48, 351) = 36.04, *p* < 0.001, *n*_*p*_^2^ = 0.48. As shown in Fig. 1, participants reported feeling vibration more intensely than sound until the seventh step, at which point they did not perceive an increase in intensity with vibration. In contrast, when using sound, each subsequent step was perceived as more intense, except for the last step. There was an inversion in the last stimulus, which did not differ from the eighth step. This result also suggests an absence of differences from the eighth stimulus in the acoustic modality.

Best fits^1^ for the estimations on the intensity levels were linear fits (Sensation_Sound_ = 8.76 × Intensity_Sound_ − 5.13; Sensation_Vibration_ = 7.38 × Intensity_Vibration_ + 3.78), with high adjustment for both modalities (Sound: *r*^2^ = 0.98; Vibration: *r*^2^ = 0.95). However, there were significant differences in the slopes of the functions, *t*(39) = 7.01, *p* < 0.001,(Sound slope = 8.76; Vibration slope = 7.38), due to the previously mentioned flat estimations in the eighth to tenth levels for the vibration dimension and the inversion on the sound dimension. In fact, the slope differences were non-significant when similar fits were estimated for the first eight stimuli of each dimension, *t*(39) =  − 0.35, *p* = 0.73.

### Discussion

In this first experiment, participants were asked to perform a psychophysical magnitude estimation task, in which they associated a level of sensation to 10 stimuli of increasing intensity using two sensory modalities. Results showed a strong significant effect of the intensity factor, with a higher intensity leading to a larger level of sensation. Additionally, there were equivalent estimations between the two sensory modalities, with both being perceived with an equivalent level of sensation on average. However, the interaction was significant, and the analysis showed that participants felt the vibration more intensely than sound until the seventh step, when they did not feel that the vibration continued to increase in intensity. On the contrary, when they used sound, each following step was perceived as more intense, except in the last step. However, in this case results show an inversion for the last two magnitudes.

Linear fits on the intensity levels showed higher adjustments that logarithmic and power functions for both modalities. These results suggest that participants were able to discriminate changes in stimulus intensity in each sensory modality with linear increments of the perceived magnitude, although there were differences in the slopes of the functions for each modality. However, results show a compression in the estimations for the higher magnitudes of both modalities. Nonetheless, the lack of discrimination for more intense stimuli can be overcome by using an accelerated transfer function in the sensorimotor task.

## Experiment 2: grasping task using sound and/or vibration

After demonstrating that intensity levels were distinguishable for both sensory modalities, and that there were equivalent estimations between them, the next step in our study was to compare the two modalities in a sensorimotor task, while maintaining consistency in the experimental setting and motor components.

The aim of Experiment 2 was to evaluate the suitability of different sensory modalities in terms of performance for grasping tasks using a SSD. Additionally, it was important to determine whether combining both sensory modalities in a SSD would have a detrimental effect on performance, as reported in Jicol et al. (2020). Based on the theories of Loomis et al. (2012) and Lenay et al. (2003), the primary hypothesis of this study was that there would be an equivalent performance between the different sensory modalities, as long as the same information and sensorimotor contingencies were provided. A secondary hypothesis was that if there were no significant performance differences between the sensory modalities, it would be possible to combine them in a SSD without detrimental effects on performance.

### Method

#### Participants

Thirty participants (23 females) with an average age of 19.85 years (SD = 2.09) were recruited. All participants were right-handed and had no prior experience with SSDs or participation in Experiment 1. The participants were undergraduate psychology students who received course credit for their participation. Prior to the experiment, all participants provided written informed consent, which was approved by the institutional ethics committee.

#### Apparatus

The same apparatus as De Paz et al. (2023) was utilized in the present study. The experiment involved grasping cylindrical plastic objects (10 cm in height) placed on a wooden table (140 cm in width, 80 cm in depth, and 75 cm in height) using only the SS glove. To limit movements to the right hand only, the left hand was positioned underneath the table, and the participant's head-trunk was fixed by placing the head to a chinrest.

A detailed description of the SS glove can be found in the methods section of Experiment 1. However, in the present experiment, the intensity of stimulation was contingent upon the real-time distance to the object, as opposed to being calculated a priori in Experiment 1. The modulation of intensity was achieved through the use of a quadratic equation, which increases the stimulation intensity at shorter distances (i.e., higher levels of intensity). The equation used was as follows:

1 $$Intensity=A\cdot {distance}^{2}+B\cdot distance+C$$

The parameters for the vibrotactile stimulation were *A* = 0.009, *B* =  − 0.5, and *C* = 10, while the parameters for the sound stimulation were *A* = 0.0507, *B* =  − 2.8182, and *C* = 83.88. The intensity range was the same as in Experiment 1, with the vibration ranging from 3.1 to 10 V, and the sound being a pure tone of 200 Hz ranging from 45 to 83.88 dB.

**Fig. 2:**
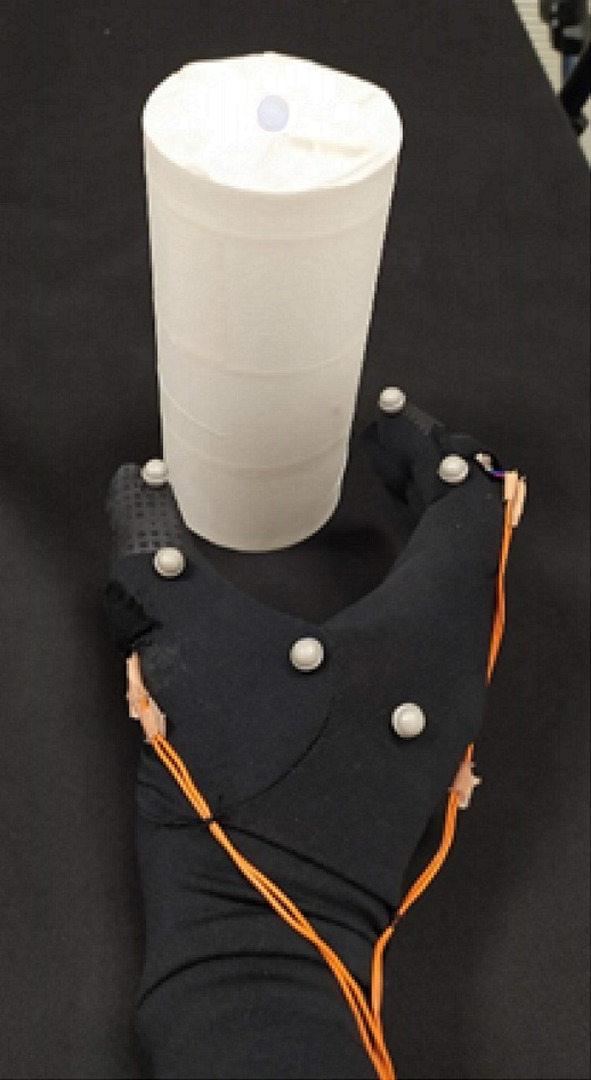
Picture of the sensory substitution glove. *Note*. Picture of the sensory substitution glove. The device consists of a regular polyester glove with six reflective markers (white semispheres) and two vibrotactile coin motors (one on the index finger and one on the thumb) which were inside a pocket

A six-camera infrared motion-capture system (MOCAP) (Qualisys AB, Göteborg, Sweden) was used to track hand movements by detecting six reflective markers located on the hand. Additionally, the position of the object was measured through a reflective marker placed on the top of the object (see Fig. 2). Hand position data was sent in real-time to MATLAB via the MOCAP system. A self-developed routine in MATLAB was used to provide contingent stimulation (in the form of sound and/or vibration, depending on the condition and distance to the object) to the index finger and/or thumb when they pointed toward the object. Additionally, the program would automatically terminate the trial if the reflective marker on the top of the object was displaced, indicating interaction with the object.

#### Design

**Fig. 3:**
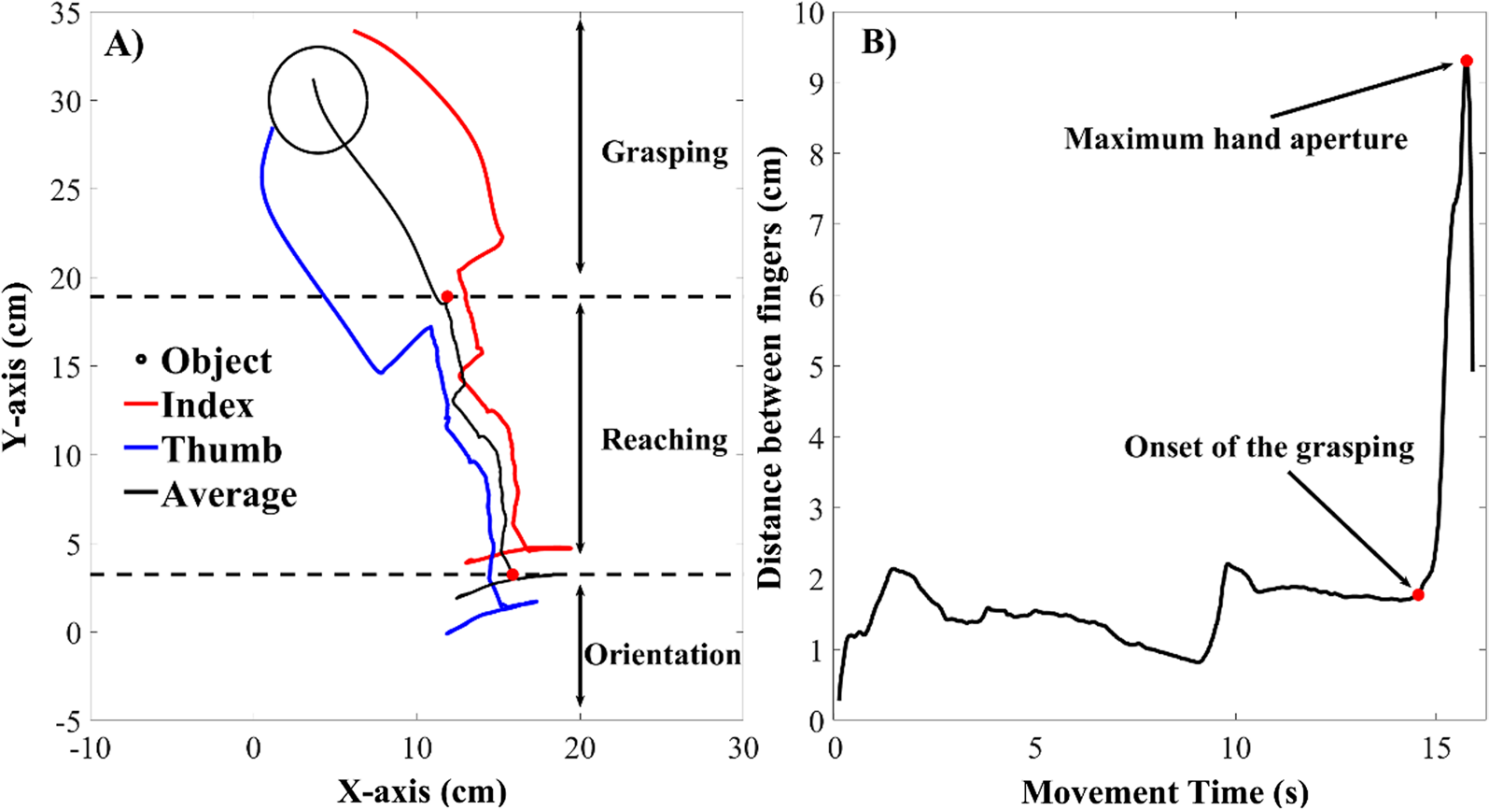
Example of a successful trial. *Note*. Example of a successful trial using only acoustic stimulation is presented. In Panel **A**, the displacement of the index finger (red line), the thumb (blue line), and the average trajectory (black line), in the *x*–*y* coordinates. Panel **B** illustrates the temporal evolution of the distance between index finger and thumb during the trial. The onset of grasping and the maximum hand aperture were highlighted with red dots. Moreover, the onset of the was used to split the reaching from the grasping phase

The present experiment employed the following within-subject factors: (1) diameter of the objects, with three levels, 4 cm, 6 cm and 8 cm; (2) three distances at 20 cm, 25 cm and 30 cm; and (3) orientation of the object in relation to the initial position of the hand, with three levels at 90°, 120° and 150°. Each condition was repeated three times, resulting in a total of 81 experimental trials per participant (3 diameters × 3 distances × 3 orientations × 3 repetitions), and their order was randomized. Due to the large number of conditions and repetitions, the sensory modality was included as a between-subject factor, with three conditions: sound, vibration, and multimodal (sound and vibration). The dependent variables measured were the proportion of correct grasps, the maximum aperture of the hand, the movement time and the proportion of time dedicated for the reaching and grasping phases. As observed in De Paz et al. (2023), participants exhibited qualitatively distinct phases during their attempt to grasp the object. We differentiated the reaching from the grasping phase by identifying the onset of the hand aperture until the maximum distance between the index finger and the thumb (see Fig. 3).

#### Procedure

Participants were blindfolded and instructed to grasp the object using only the SSD, with no time or hand movement constraints, and without touching the cylinder prior to the trial. They were informed of the specific version of the SSD they would be using. Prior to each trial, the participant's hand was placed at the initial position, and the trial automatically terminated when the participant touched the cylinder. The researcher recorded the outcome as a correctly grasped object when it was grasped and lifted with the hand. To familiarize participants with the task, prior to the experimental trials, participants completed eight familiarization trials using two objects of 5 cm or 7 cm in diameter, two distances of 22 cm or 28 cm, and two orientations of 105° or 135° using the specific device modality. Participants were blindfolded throughout the experiment, and the entire session lasted one hour. After the experiment, participants who performed the multimodal condition completed a short questionnaire (see Appendix 1) which included questions about the sensory modality they primarily used at the beginning, middle and end of the trials, the perceived usefulness of each sensory modality, and which sensory modality they would choose for a SSD in their daily life.

#### Data acquisition, preprocessing and statistical analysis

We captured the trajectory of the hand during each trial and focused specifically on the hand movements in the horizontal plane. The *y*-axis was defined as the direction from the starting position of the hand to the target, while the *x*-axis was established as being perpendicular to the *y*-axis. It should be noted that the coordinate system varied for each position of the target. To further analyze the data, a 4th-order low-pass Butterworth filter with a cutoff frequency of 8 Hz was applied. We averaged the data over repetitions and performed two analyses. First, we conducted an equivalence Bayesian test for independent samples to test whether the sensory modality groups were significantly non-different from each other. Second, we carried out a mixed ANOVA after averaging the data over repetitions. Movement variables were only analyzed in those trials in which the object was correctly grasped. When the assumption of sphericity was violated, Greenhouser-Geisser corrections were employed. As a post-hoc test, Bonferroni corrections were utilized, and the level of significance was set at *α* = 0.05.

### Results

As illustrated in Fig. 3, a visual representation of a successful trial exhibits the various stages of the movement. Participants initially engage in radial movements to locate the object and orient their hand toward it. Subsequently, they move their hand toward the object and, as they approach it, initiate the grasping phase by increasing the aperture between their fingers until contact with the object's surface.

#### Proportion of correct grasps

In overall, the object was correctly grasped in 0.84 of the trials. According to the equivalence test, there were strong evidence (Table 2) in favor of equivalence between the sensory modality groups (Sound: *M* = 0.83, SD = 0.21; Vibration: *M* = 0.85, SD = 0.21; Multimodal: *M* = 0.85, SD = 0.20). The mixed ANOVA showed that neither the within-subject factors nor their interactions were significant. Participants grasped the objects without significant differences in relation to their diameter, distance, or orientation.

**Table 2:** Bayes factor (BF_10_) for each dependent variable for each pair of sensory modality groups

	Sound versus vibration	Sound versus multimodal	Vibration versus multimodal
P (correct)	12.111	9.462	14.667
Max hand Ap	4.352	3.393 × 10^–10^	1.444 × 10^–8^
Mov. Time	1.896 × 10^–12^	1.438 × 10^–11^	0.016
P. Time reach	1.578 × 10^–11^	1.399 × 10^–11^	0.564

We use the interpretation of BF_10_ values by van Doorn et al. (2021)

#### Maximum aperture of the hand

The equivalence test revealed that there was very strong evidence to support that the multimodal group was the only non-equivalent group (Table 2). The multimodal group performed a lower maximum hand aperture (M = 8.20 cm, SD = 3.10) than the rest of the groups (Sound: M = 9.54, SD = 2.90; Vibration: M = 9.27, SD = 2.93). The mixed ANOVA revealed that the diameter factor had a significant effect, *F*(1.75,216) = 4.78, *p* < 0.05, *n*_*p*_^2^ = 0.15, on the maximum hand aperture. Specifically, the Bonferroni post-hoc test showed that the wider object (8 cm) differed significantly from the smaller cylinder (*p* < 0.05) (8 cm: *M* = 9.25 cm, SD = 2.00; 6 cm: *M* = 8.89 cm, SD = 2.17; 4 cm: *M* = 8.88 cm, SD = 2.46). Additionally, the orientation factor had a strong significant effect, *F*(1.25,216) = 277.91, *p* < 0.001, *n*_*p*_^2^ = 0.91. The participants opened the hand wider as the object was placed more perpendicular to their position (*p* < 0.05) (90º: *M* = 10.72 cm, SD = 2.24; 120º: *M* = 9.29 cm, SD = 2.18; 150º: *M* = 7.00 cm, SD = 2.21). The interaction between orientation and distance was significant, *F*(3.6,216) = 3.21, *p* < 0.05, *n*_*p*_^2^ = 0.11. When the object was placed at 90° and at 25 cm, the participants performed a significantly smaller hand aperture (*M* = 6.74 cm, SD = 2.16) than for the rest of the conditions (*p* < 0.05). The sensory modality was not significant, but the interaction between the diameter and the sensory modality group was significant, *F*(3.5,216) = 3.38, *p* < 0.05, *n*_*p*_^2^ = 0.20. The participants who performed the experiment with sound and vibration performed a significantly larger hand aperture for the larger object (8 cm: *M* = 8.77, SD = 2.19; 6 cm and 4 cm: *M* = 7.86 cm, SD = 2.23) than for the rest of the cylinders (*p* < 0.01).

#### Movement time

There was very strong evidence to support that the sound group was significantly non-equal than the other groups. Besides, the multimodal group was anecdotally different from the vibration group (Table 2). In other words, the sound group needed 15.88 s (SD = 8.88) to perform the task, followed by the multimodal group (*M* = 12.52 s, SD = 5.9) and then by the vibration group (*M* = 11.16 s, SD = 7.01). The results of the mixed ANOVA indicated that all within-subject factors had a significant effect on the grasping time. Specifically, the orientation factor had a significant effect, *F*(1.5,216) = 25.32, *p* < 0.001, *n*_*p*_^2^ = 0.48, showing that as the object was positioned more perpendicular to the participants, they required more time to grasp the object (*p* < 0.01; 90º: *M* = 14.21 s, SD = 5.84; 120°: *M* = 13.12 s, SD = 5.78; 150°: *M* = 11.36 s, SD = 5.56). Additionally, the distance factor had a significant effect, *F*(1.5,216) = 162.18, *p* < 0.001, *n*_*p*_^2^ = 0.86, grasping time increased as the distance to the object increased (30 cm: *M* = 15.84 s, SD = 6.98; 25 cm: *M* = 12.55 s, SD = 5.59; 20 cm: *M* = 10.30 s, SD = 4.60). Furthermore, the diameter factor also had a significant effect, *F*(1.7,216) = 28.50, *p* < 0.001, *n*_*p*_^2^ = 0.51, with a shorter grasping time for smaller diameter objects (8 cm: *M* = 11.63 s, SD = 5.13; 6 cm: *M* = 12.79 s, SD = 5.60; 4 cm: *M* = 14.27 s, SD = 6.45). The only significant interaction was between the distance and sensory modality factors, *F*(3.0, 216) = 7.30, *p* < 0.001, *n*_*p*_^2^ = 0.35. This interaction revealed that within the objects placed at 30 cm, the group that performed the experiment with sound required significantly more time than the group that used vibration (*p* < 0.05; Sound: M = 19.31 s, SD = 6.72; Vibration: M = 12.90 s, SD = 5.99).

#### Proportion of time until the onset of the grasping phase

Overall, participants spent an average proportion of 0.79 (SD = 0.12) of the trial locating and displacing their hand toward the object (localization and reaching phases). The equivalence test showed, with strong evidence, that the sensory modality groups were not equal (Table 2). The multimodal group started the grasping phase earlier (*M* = 0.77; SD = 0.14) than the vibration group (*M* = 0.79; SD = 0.16) and followed by the sound group (*M* = 0.85; SD = 0.13). A mixed ANOVA revealed that only the distance factor had a significant effect, *F*(2, 54) = 12.99, *p* < 0.001, *η*_*p*_^2^ = 0.32. Post-hoc comparisons indicated that participants performed the grasping phase significantly earlier (*p* < 0.001) when the object was placed at 20 cm (*M* = 0.77, SD = 0.13) compared to the other distances (*M* = 0.80, SD = 0.11). None of the interactions were significant.

#### Survey

**Fig. 4:**
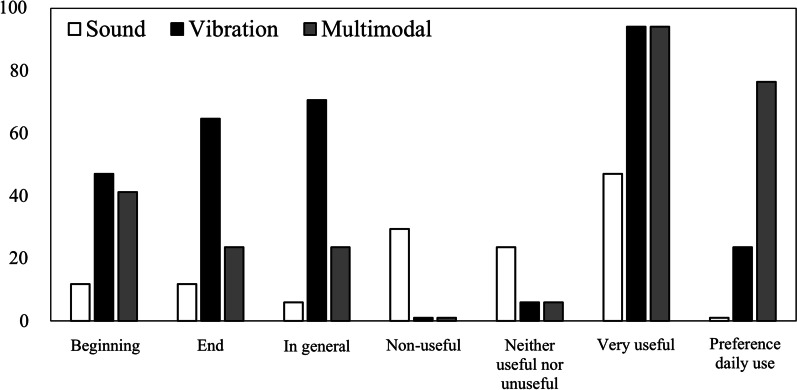
Summary of the survey. *Note* Percentage of affirmative answers to the survey questions by participants in the multimodal condition

Those participants who performed the experiment using the multimodal condition completed a form (Appendix 1). The results can be seen in Fig. 4. Vibration was the most utilized modality among the participants in the multimodal condition (70%). This was followed by the combination of vibration and sound (20%), and sound alone (10%). Additionally, the participants reported that sound was not particularly useful for solving the task, and none of them stated they would use an acoustic version of the glove in their daily lives. In contrast, many participants reported that the combination of both sources of stimulation was useful, although they did not use them equally. Furthermore, a majority of participants expressed a preference for a multimodal device for use in their daily lives.

### Discussion

In this second experiment, we compared the effectiveness of sound and vibration in a grasping task including variations on diameter, distance, and orientation, of cylindrical objects. Participants were guided by vibrotactile, acoustic, or a combination of both sensory modalities in a multimodal condition. Our results showed that overall, the objects were correctly grasped in 84% of the trials. This finding is consistent with previous research by De Paz et al. (2023), which confirms the potential of this SS glove for grasping objects. Notably, we did not observe any significant effect of the object-related factors on performance. Regardless of the diameter, distance and orientation of the object, participants always grasped it with the same success rate under the different sensory conditions. These results suggest that both vibration and sound are equivalently effective for use in SSDs when transmitting gradient or magnitude information (Loomis et al., 2012). Although the multimodal condition did not significantly increase the proportion of correct grasps, it did not result in any decrements in performance as observed in other studies (Jicol et al., 2020). This suggests that decrements in performance previously observed may be attributed to the specific information provided to the different modalities, rather than an intrinsic problem with multimodal integration.

Concerning the movement variables, participants reproduced the main patterns observed on previous research on grasping. As it can be expected, the aperture of the hand was found to vary significantly with the diameter of the to-be-grasped objects. However, it was observed that the relative aperture was larger for smaller objects. This result is consistent with previous research in which grasping was performed without visual control or with perturbations in object properties (Castiello et al., 1993; Chieffi & Gentilucci, 1993; Jakobson & Goodale, 1991; Marteniuk et al., 1990; Mon-Williams et al., 2001; van de Kamp & Zaal, 2007; Wing et al., 1986; Zaal & Bongers, 2014; Zaal et al., 1998). This phenomenon can be related to a safety strategy (Kolarik et al., 2016). As smaller objects are known to be more difficult to be grasped than larger ones (Smeets & Brenner, 1999), this strategy maximizes performance and was also valid, as participants were not instructed on how to grasp the objects. Additionally, the orientation of the object was found to significantly influence the aperture of the hand during grasping. The more perpendicular the cylinder was, the greater the hand opening. This effect can also be related to a safety strategy, as perpendicular positions typically present more difficulties than other polar positions.

The results of this study indicate that movement time was significantly affected by various factors, including the distance of the object, its diameter and orientation. This is consistent with previous research on visually controlled grasping, which has shown that movement time is sensitive to object properties (Paulignan et al., 1997). The last movement variable analyzed was the proportion of the time spent until the onset of the grasping phase. Our results showed a significant effect of the distance to the object. That is, the localization and reaching phases lasted longer the further away the object was. These findings demonstrate that participants were able to detect the properties of objects prior to physically interacting with them and were able to adapt their grasping strategies accordingly.

However, significant differences in movement variables were observed across sensory modalities. Notably, the maximum hand aperture, a measure often linked to the perception of object size in the literature, was significantly shorter in the multimodal condition than in the two unimodal conditions. Moreover, we observed a significant interaction effect with the diameter, indicating that the hand aperture was larger for the acoustic condition when exploring smaller diameter objects. These findings suggest that participants exhibited greater confidence in their perception of object size in the multimodal condition, possibly as a result of receiving redundant information from both sensory modalities.

Similar results were observed with the proportion of time until the onset of the grasping phase, which can be related to the perception of the distance to the object. Again, the proportion of time spent until the onset of the grasping phase was significantly lower for the multimodal condition followed by the vibrotactile condition, and finally by the acoustic condition. In addition, there was an increase in the proportion of time until the grasping started for the acoustic condition when the object was farther away. In line with the safety margin hypothesis (Kolarik et al., 2016), our findings suggest that the multimodal condition elicited greater confidence and precision than the vibrotactile condition, and that the vibrotactile condition produced more confidence and precision than the acoustic one for perceiving the distance to the object. Finally, although movement time is a holistic movement variable that encompasses several factors, including the three phases of the grasp function (De Paz et al., 2023), it can be interpreted as an indicator of feedback reliability in the task. In this sense, the task was performed correctly requiring the shortest movement time in the vibrotactile condition, followed by the multimodal condition and finally by the acoustic condition.

These results are consistent with the survey reports, in which participants consistently rated vibration as the most useful modality for all phases of the task, while some considered the acoustic condition to be non-useful. Most participants expressed a preference for the multimodal version for daily use. However, it is important to note that overall performance, measured by the proportion of correct grasps, was equivalent in the three sensory modalities.

## General discussion

In this section, we aim to discuss the significance of substituting modalities and the phenomenon of multisensory integration in the context of sensory substitution. Loomis et al., (2012, 2013) proposed that functional equivalence can be achieved in SSDs when gradient or magnitude information is transmitted across sensory modalities with equivalent bandwidth for the continuum. On the other hand, Lenay et al. (2003) suggested that equivalent sensorimotor contingencies lead to functional equivalence between sensory modalities. Our study meets the criteria of similar sensorimotor contingencies and equivalence between sensory modalities in the transmission of magnitude information. Therefore, our results showing that no sensory modality consistently outperforms the others are in coherence with the previous functional equivalence predictions.

Additionally, we have demonstrated that unimodal stimulations can be combined in SSDs (Lloyd-Esenkaya et al., 2020), as previously demonstrated in other studies (Buchs et al., 2017; Hoffman et al., 2018; Maidenbaum et al., 2014b). These results suggest that the incompatibility between sensory modalities observed in other studies (Jicol et al., 2020; Richardson et al., 2019) may be more a result of incompatibility between devices and/or the information provided, rather than due to sensory systems. This is also consistent with Loomis et al. (2012), who highlighted the need to analyze whether the to be provided information by the device requires higher-order cognitive operation.

Despite we found an overall equivalent performance between unimodal and multimodal conditions, this does not necessarily indicate that multisensory integration has been achieved in the multimodal condition. Some theories of multimodal integration would support an improvement of performance when there is redundant information (Mayer & Johnson, 2008). At the same time, it would be possible to solve the task using only one sensory modality, while ignoring the other (Stein, 1998; Stein & Wallace, 1996; Stein et al., 2010). In fact, the analysis of the movement variables has shown differences in hand aperture and the proportion of time spent before the grasping phase that seems to indicate better movement performance by the multimodal over the unisensory conditions. Moreover, the proportion of time until the onset of the grasping phase was also significantly lower for the vibrotactile vs. the acoustic condition, which also suggests a better detection of distance through vibration than sound.

The results of the survey indicate that the majority of participants preferred to use vibrotactile stimulation, with many participants ignoring sound as they found it less useful in the multimodal condition. Furthermore, none of the participants reported that they would use an SSD that only utilized sound in their daily life. This may suggest that the format used for acoustic stimulation was not optimal, and that users may have preferred other properties of the sound signal. For future research, it would be interesting to compare the performance obtained with different sound properties, such as changes in pitch as a function of the distance to the object. In addition, whereas the vibrotactile stimulation was delivered to the hand, the sound was delivered through the ears. Therefore, it might be easier to stablish the sensorimotor contingencies under the vibrotactile condition. To control that possibility, future research would test different body locations for vibrotactile stimulation. The multimodal version of the glove was rated as useful as the vibrotactile version and the most preferable sensory modality by participants. This level of preference, combined with the low reported use of sound, suggests that the sensory modalities did not have equal relevance. In our study, touch was the most utilized sensory modality, although participants preferred to add the acoustic stimulation to the SSD. Although previous research has shown that the grasping behavior of people with visual impairments does not significantly differ from that under visual control once the object is located in space, future research should test the device in this population, as they are the primary target of these devices.

Integrating two sensory modalities in an SSD is not an extended practice in SSD research, but multisensory integration is a continuous process in our daily lives. In other words, it is difficult for isolated perceptual experiences to occur in a natural context, as perception is usually the result of the combination of different sources of stimulation such as vision, sound, proprioception, vestibular, etc. (Jicol et al., 2020; Lloyd-Esenkaya et al., 2020; Stein et al., 2010). The amodal conception of perceptual information (Lenay et al., 2003; Stoffregen & Bardy, 2001) and theories of mental amodal representation (Loomis et al., 2012, 2013; Mayer & Johnson, 2008) provide a theoretical basis for the integration of multiple perceptual modalities in SSDs. Our study demonstrates that if these principles are meet, the SSD will enable users to successfully perform the specific tasks.

## Conclusions

To conclude, we would like to emphasize the high level of performance achieved by inexperienced users with the device. After a few trials, users demonstrated a great ability to perform a complex sensorimotor task such as detecting different object properties and adapting their movement patterns to perform a grasp. We believe that if such a short familiarization period can achieve such good results, an appropriate training program would produce a substantial improvement in their performance. Additionally, we have shown that, although there are differences in the movement patterns, both vibration and sound, and their multimodal integration are suitable sensory modalities to use with our SSD. All of these elements, coupled with improvements in the technological aspect of the device, would bring us closer to the ultimate goal of widespread use of sensory substitution devices.

## Data Availability

The datasets used and/or analyzed during the current study are available from the corresponding author on reasonable request.

## Appendix 1

See Table 3.

**Table 3:** Survey fulfilled by participants who performed Experiment 2 in the multimodal condition

	I only used sound	I used sound more than vibration	I used both modalities equally	I used vibration more than sound	I only used vibration
At the beginning of the trial					
At the end of the trial					
During the trial					
How informative did you find it?	Very useless	Somehow useless	Neither useful nor useless	Somehow useful	Very useful
Sound					
Vibration					
Multimodal					
		Sound	Vibration	Multimodal	
If you were to use this device daily, what type of sensory modality would you like to use? (Choose only one)					

## Abbreviations

SSD: Sensory substitution devices
SoV: Sound of vision
MOCAP: Motion-capture

## Footnotes

1 Power fits adjustments (Sound: *r*^2^ = 0.9792; Vibration: *r*^2^ = 0.9471). Log fits adjustments (Sound: *r*^2^ = 0.9434; Vibration: *r*^2^ = 0.8758).
